# Comparison of VIDAS and Radioimmunoassay Methods for Measurement of Cortisol Concentration in Bovine Serum

**DOI:** 10.1155/2013/216569

**Published:** 2013-10-29

**Authors:** Daniela Proverbio, Roberta Perego, Eva Spada, Giada Bagnagatti de Giorgi, Angelo Belloli, Davide Pravettoni

**Affiliations:** Dipartimento di Scienze Veterinarie per la Salute, la Produzione Animale e la Sicurezza Alimentare, Università degli Studi di Milano, Via Celoria 10, 20133 Milan, Italy

## Abstract

Radioimmunoassay (RIA) is the “gold standard” method for evaluation of serum cortisol concentration. The VIDAS cortisol test is an enzyme-linked fluorescent assay designed for the MiniVidas system. The aim of this study was to compare the VIDAS method with RIA for measurement of bovine serum cortisol concentration. Cortisol concentrations were evaluated in 40 cows using both VIDAS and RIA methods, the latter as the reference method. A paired Student's *t*-test, Pearson's correlation analysis, Bland-Altman plot, and Deming regression analysis were used to compare the two methods. There was no statistically significant difference between mean serum cortisol concentrations measured by VIDAS or RIA methods (*P* = 0.6570). Both methods were able to detect significant differences in mean low and high cortisol concentrations (*P* < 0.00014 RIA and *P* < 0.0016 VIDAS). The correlation coefficient was low, but a Bland-Altman plot and Deming regression analysis show neither constant nor proportional error. The VIDAS method produced slightly higher values than RIA, but the difference was small and in no case did the mean value move the normal range. Results suggest that VIDAS method is suitable for the determination of bovine serum cortisol concentration in studies of large numbers of animals.

## 1. Introduction

Cortisol is involved in numerous metabolic and immunologic functions. Serum cortisol concentration varies due to circadian rhythms, diet, environmental temperature, or humidity and physiological conditions [1, 2]. In farm animals, measurement of serum cortisol concentration has been used in the assessment of stress and pain caused by mismanagement, travel, inappropriate environmental temperature, castration without local anesthesia, and disease [2–7]. Measurement of serum cortisol concentration is common in large animal medicine to monitor effects of modern farming practices on animal welfare [1]. Radioimmunoassay (RIA) is the traditional “gold standard” method for evaluation of serum cortisol concentration [8, 9]. However, there are several disadvantages of this method such as short shelf-lives of the radioactive reagents, risk of radiation exposure for staff, and the need to dispose of toxic waste [8, 10, 11]. In recent years, several alternative nonradioactive techniques have been developed for the measurement of cortisol concentrations in animals, including chemiluminescent and enzyme immunoassays [8–10]. The VIDAS cortisol test is an enzyme-linked fluorescent assay (ELFA) designed for the MiniVidas system. The MiniVidas is a compact, rapid, automated immunoassay analyzer that needs to be calibrated only once every 14 days, optimizing the per result cost. Tests sharing similar protocols may be run together in 1 section of the analyzer and each section functions independently from the other. Moreover, it is possible to perform a single test with single dose reagents. Whilst RIAs are usually used only in specially equipped laboratories, the MiniVidas is cheaper and well suited for routine work. The MiniVidas has been successfully used to measure the concentration of several human hormones including insulin, human chorionic gonadotropin, progesterone, and cortisol. [12, 13]. Its use has already been validated in the dog [14]. The aim of this study was to compare the VIDAS ELFA assay with the RIA immunoassay for measurement of bovine serum cortisol concentration.

## 2. Materials and Methods

### 2.1. Animals and Analytic Procedures

In order to mimic the situation in clinical practice, animals were selected on the basis of a clinical status that would be expected to be characterized by either normal or high serum cortisol concentrations. Blood samples were collected from 29 “downer” cows admitted to the Clinic for Ruminants and Pigs of the Large Animal Teaching Hospital of Lodi. Animals ranged from 2.5 to 9 years of age (mean ± SD, 4 ± 2 years) and body weight ranged from 450 to 850 kg (mean ± SD, 560 ± 160 kg). Twenty-one animals were in late pregnancy or had just calved (±20 days). In addition, 11 healthy dairy cows ranging from 3 to 6 years old in the 2nd–4th week postpartum were selected from 2 herds located in Lombardy (Italy). The healthy cows had a milk production level of 20–40 liters a day and were fed a diet of corn and grass silage with hay, supplemented with concentrate according to the milk production level.

In both the healthy controls and “downer” cows surplus serum from samples submitted for routine biochemical and hematological profiles was used. Blood samples were collected from the jugular vein in Vacuette tubes (Greiner BIO-ONE GmbH, Kremsmunster, Austria). Samples were immediately centrifuged, and the serum was removed and divided into 2 aliquots that were stored at −20°C until analysis were performed within 15 days. Immediately after thawing, cortisol was measured by both radioimmunoassay and by VIDAS assay (1 aliquot per method) in a single run. All aliquots were subjected to exactly the same handling procedures before analysis.

### 2.2. Cortisol Titration by Radioimmunoassay

The radioimmunoassay (RIA) method used for cortisol titration was a competitive solid phase radioimmunoassay validated for cattle (RIA—competitive solid phase radioimmunoassay—Coat-a-Count Cortisol, Siemens, Germany) [15, 16]. Cortisol concentrations are typically low in cattle, so to obtain the calibration curve 2 uncoated 12 × 75 mm polypropylene tubes *T* (total counts) were labeled in duplicate, and 12 Cortisol Ab-Coated tubes A (maximum binding) and B to F were also labeled in duplicate. A 100 *μ*L aliquot of the zero calibrator A was pipetted into the A tubes and 75 *μ*L of the zero calibrator A into the remaining calibrator tubes B to F. Finally 25 *μ*L of each control B to F was pipetted into the correspondingly labeled tubes so that each calibrator tube contained 100 *μ*L. The NSB tubes were omitted, as suggested by manufacturer's instructions. Finally 1 mL of ^125^I-cortisol was added to each tube, and the tubes were centrifuged and then incubated for 90 minutes at 37°C in a water bath. The supernatant was aspirated and the tubes analyzed in a gamma counter to obtain the calibration curve. After thawing and gentle swirling of each plasma sample from the study, 100 *μ*L of plasma was placed into the duplicate coated tubes, to which 1 mL of ^125^I-cortisol was added and then centrifuged. The tubes were incubated for 90 minutes at 37°C in a water bath. The supernatant was aspirated and the tubes analyzed in a gamma counter. This solid phase RIA has an analytical sensitivity of 5.5 nmol/L and a calibration (working) range of 14–1380 nmol/L [17]. In healthy cows cortisol concentrations measured by RIA range from 6.74 to 56.30 nmol/L. Normal values were established for our laboratory by use of samples obtained from 50 clinically normal, lactating dairy cows 3 weeks after parturition. This assay was used as the reference method in the study.

### 2.3. Cortisol Titration by VIDAS Assay

Cortisol concentrations were measured using both the VIDAS assay and an automated test for the quantitative determination of cortisol in human serum, on the MiniVidas analyzer (BioMerieux S.A., Lyon, France). According to the manufacturer the assay has a measurement range of 5.51–2759 nmol/L for human serum. Both analyzers were cleaned, calibrated, and operated in accordance with the manufacturer's instructions. To establish the intra-assay variation of the VIDAS method, 2 bovine samples with a high (217.7 nmol/L) and low (22.4 nmol/L) cortisol concentration were analyzed 10 times on the same day [16]. The coefficient of variation (CV) was calculated as SD/mean × 100. Acceptance limits based on the inherent imprecision of both methods were calculated by calculating the value twice and then using the mean of these calculations in the formula. Maximum allowable values for imprecision were obtained from desirable specification for total error, imprecision, and bias derived from biological variation in people [18]. Interassay variability was estimated by determining cortisol concentrations in the same 2 (high and low cortisol concentration) bovine serum samples. Duplicate analyses were run twice each day for 5 days. The CV was calculated. To establish the linearity of the new method, a sample with a high cortisol concentration (217.7 nmol/L) was assayed in duplicate following various dilutions with 0.9 g/L NaCl.

### 2.4. Statistical Analysis

Statistical analyses were performed using MedCalc for Windows (version 11.5.1, MedCalc Software bvba, Mariakerke, Belgium). The Kolmogorov-Smirnov test was performed to determine whether the data was normally distributed. Descriptive statistics (mean, median ± standard deviation, range) were assessed for concentrations measured using RIA and VIDAS methods. A paired Student's *t*-test was used to test for significant differences between overall mean cortisol results obtained by VIDAS and RIA methods. Cortisol concentrations obtained by RIA were compared using paired Student's *t*-tests to assess the difference in cortisol concentration in healthy or downer cows. Results were reported as mean ± SD.

Paired Student's *t*-test was used to evaluate differences between cortisol results in healthy cows evaluated by RIA and VIDAS methods. The same paired Student's *t*-test was used to evaluate differences between cortisol results in downer cows evaluated by RIA and VIDAS methods. Statistical significance for all tests was defined as *P* < 0.05.

Correlation of cortisol values obtained by the VIDAS method relative to values obtained by the RIA reference method was compared using Pearson's correlation analysis and simple regression analysis [19]. Agreement between the 2 methods was calculated with a Bland-Altman plot, where bias is defined as the mean difference between methods, and with Deming regression analysis. Agreement was considered to be good if the 95% confidence interval (CI) of the intercept and slope from the Deming regression included 0 and 1, respectively [20–22].

To assess the ability of the VIDAS method to detect high cortisol serum concentration in cattle, the sensitivity and specificity were calculated using RIA results as true positive values.

## 3. Results

There was no statistically significant difference between serum cortisol concentrations in overall samples measured using VIDAS and RIA methods (*P* = 0.6570, Student's paired *t*-test) (Table 1). However, cortisol concentrations obtained using the VIDAS method were 9.96% higher than those obtained with RIA (mean RIA 124.88 nmol/L, mean VIDAS 137.33 nmol/L). Mean cortisol concentration obtained by RIA was significantly different in healthy cows compared to downer cows (*P* < 0,0014).

There was no significant difference between mean cortisol concentration measured using the VIDAS or RIA methods in the healthy cows group (*P* = 0.3575). The same result was obtained in the downer cows group (*P* = 0.7063).

**Figure 1:**
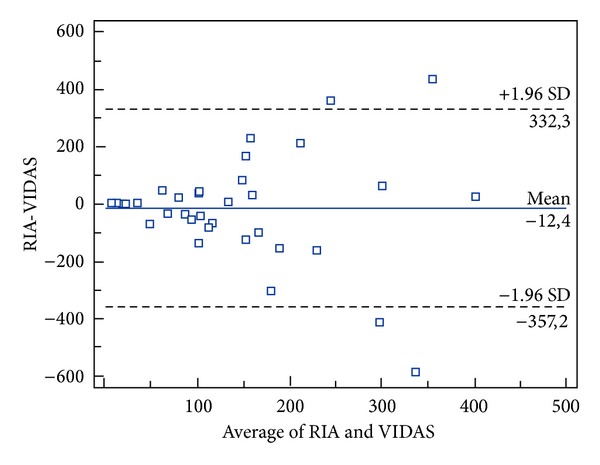
Comparison between the VIDAS assay and the RIA reference method for cortisol serum concentration (nmol/L) in 40 cows Bland-Altman plot. The solid line indicates the mean of difference, and dashed lines indicate limits of agreement, which are defined as the mean difference plus and minus 1.96 times the standard deviation of the differences.

Both methods were able to detect significant differences in mean cortisol concentrations between the healthy and downer cow groups (*P* < 0.00014 RIA and *P* < 0.0016 VIDAS independent samples *t*-test). The RIA had an intra-assay variability of 8% (mean of duplicates). Intra-assay variation using the VIDAS method was 10.4% for samples with a high cortisol concentration and 9.8% for samples with a low cortisol concentration. The combined inherent CV of the 2 methods was *√*10.1^2^/2 + 8^2^/2 = 9.11%. Thus, at least 95% of the difference between the methods was expected to be within the interval 0 ± 1.96 × 9.11 = 0 ± 17.8%. The following biological variation for the maximum allowable values for imprecision was obtained from data on desirable specification for total error, imprecision, and bias derived from biological variation for cortisol in people: imprecision, *I*_max⁡_ = 10.5%; inaccuracy, *B*_max⁡_ = 12.5%; and maximum total error allowable, TE_max⁡_ = 29.8%. Recovery of cortisol was high at all dilutions (Table 2). The correlation coefficient (*r*) between cortisol values measured by the RIA and VIDAS methods was 0.1790 (95% CI −0.1403 to 0.4646). Since the *r* value was less than 0.975 alternative regression analysis, such as Deming regression, was needed when both the new and the reference method were measured with error [19]. Using Deming regression analysis the intercept −89.2631 (95% CI = −26.0478 to 204.5741) was not different from 0 and the slope 0.3849 (95% CI = −0.6745 to 1.4443) was not different from 1; hence neither constant nor proportional error were present. Acceptability based on inherent imprecision of both methods was assessed with a Bland-Altman plot. Eighteen of 40 (45%) measurements were within the interval 0 ± 17.8%; therefore, the 2 methods were not identical within inherent imprecision (Figure 1).

All serum cortisol concentrations measured in healthy cows by the RIA method were within normal limits for bovine cortisol as established for our laboratory. In 5/40 analyzed samples the two methods were discordant in discriminating normal and pathological values using a cortisol concentration of 56.3 nmol/L as a cutoff; that is, the VIDAS method incorrectly categorized 4 samples as either pathological or normal.

The sensitivity and specificity of the VIDAS method were 96% (95% CI 0.791–0.998) and 76% (95% CI 0.459–0,938), respectively.

## 4. Discussion

Human assays should not be used to analyze animal serum unless they have been adequately validated. The VIDAS analyzer has good reproducibility, precision, and specificity for determining cortisol concentrations in human serum [14]. Cortisol concentrations in bovine serum measured using the VIDAS (5–628 nmol/L) and RIA (6.9–577 nmol/L) methods were comparable in the range of cortisol concentrations seen in this study. Mean overall cortisol concentrations for the various groups were also similar for the 2 assays (RIA 124.88 nmol/L and VIDAS 137.33 nmol/L). In this study, we did not evaluate possible analytical interferences such as plasma hemoglobin, bilirubin, or lipemia, but the effects of these biochemical changes should be considered. We were able to show a weak correlation (*r* = 0.1790) between cortisol concentrations measured using the VIDAS and RIA methods, but Deming regression shows that no constant or proportional errors were present. Furthermore, the Bland-Altman test of agreement demonstrated that the VIDAS method produced results close to those obtained by the reference method, and the variation of cortisol concentrations measured using the VIDAS method seemed to be highly affected by the magnitude of cortisol concentrations measured, being more accurate at lower cortisol concentrations. The VIDAS method produced slightly higher values than RIA, but the difference was small and both mean values remained within the normal range. When a cortisol concentration, of 56.3 nmol/L was used as the cut off between normal and high serum cortisol concentration, the results obtained with VIDAS did not agree with those obtained with RIA reference method in 4 samples.

## 5. Conclusions

The MiniVidas analyzer was simple and fast to operate. The rapidity of measurement (30 minutes), small sample required (200 *μ*L), and wide working range make the VIDAS method suitable for field studies in farm animals. Results obtained in this study suggest that the VIDAS method is suitable for the determination of bovine serum cortisol concentration in studies involving large numbers of animals, in particular where the mean value of the group is relevant—such as studies on stress. On the other hand, when greater accuracy and precision are needed for clinical assessment of the individual subject, this study suggests that this method is not advisable due to its relatively low specificity (76%).

## Figures and Tables

**Table 1:** Comparison of results obtained using the VIDAS and RIA methods for serum cortisol determination in 40 cows.

Cortisol concentration (nmol/L)						
Method	Mean	SD	Mean 95% Confidence interval	Median	Minimum	Maximum
VIDAS	21.8	24.45	5.37–38.23	95.2	5	628.6
RIA	13.05	12.6	4.56–21.55	83.65	6.9	577.3

**Table 2:** Serum concentration and percentage recovery of cortisol using the VIDAS method on bovine serum with high cortisol concentration.

Dilution	Expected (nmol/L)	Observed (nmol/L)	Recovery (%)
1 : 1	217.7	217.7	100
1 : 2	108.8	105.3	97
1 : 4	54.42	65.8	79
1 : 8	27.2	22.1	81
